# LXA_4_-FPR2 signaling regulates radiation-induced pulmonary fibrosis via crosstalk with TGF-β/Smad signaling

**DOI:** 10.1038/s41419-020-02846-7

**Published:** 2020-08-08

**Authors:** Hyunjung Kim, Sung-Hyo Park, Song Yee Han, Yun-Sil Lee, Jaeho Cho, Jin-Mo Kim

**Affiliations:** 1 Department of Radiation Oncology, Yonsei University College of Medicine, Seoul, South Korea; 2 Graduate School of Pharmaceutical Sciences, Ewha Womans University, Seoul, South Korea; 3 Department of Manufacturing Pharmacy, Natural Product Research Institute, College of Pharmacy, Seoul National University, Seoul, South Korea

**Keywords:** Target identification, Respiratory tract diseases

## Abstract

Radiation therapy is an important modality in the treatment of lung cancer, but it can lead to radiation pneumonitis, and eventually radiation fibrosis. To date, only few available drugs can effectively manage radiation-induced pulmonary fibrosis. Lipoxins are endogenous molecules exhibit anti-inflammatory and pro-resolving effects. These molecules play a vital role in reducing excessive tissue injury and chronic inflammation; however, their effects on radiation-induced lung injury (RILI) are unknown. In this study, we investigated the effects of lipoxin A_4_ (LXA_4_) on RILI using our specialized small-animal model of RILI following focal-ablative lung irradiation (IR). LXA_4_ significantly inhibited immune-cell recruitment and reduced IR-induced expression of pro-inflammatory cytokines and fibrotic proteins in the lung lesion sites. In addition, micro-CT revealed that LXA_4_ reduced IR-induced increases in lung consolidation volume. The flexiVent^TM^ assays showed that LXA4 significantly reversed IR-induced lung function damage. Moreover, LXA4 downregulated the activities of NF-κB and the Smad-binding element promoters. The expression of FPR2, an LXA_4_ receptor, increased during the development of IR-induced pulmonary fibrosis, whereas silencing of endogenous LXA_4_ using an antagonist (WRW4) or FPR2 siRNA resulted in impaired development of pulmonary fibrosis in response to IR. Collectively, these data suggest that LXA_4_ could serve as a potent therapeutic agent for alleviating RILI.

## Introduction

Radiation therapy is an important modality for treating lung cancer. Generally, it is necessary to increase the radiation dose to control the tumor by radiation, but higher radiation dose results in higher probability of developing side effects. Therefore, radiation-induced lung damage (RILI) is a major bottleneck when aiming to improve disease-free survival and well-being in patients with lung cancer^1–3^. RILI is classified into two types, acute phase radiation pneumonitis—that generally subsides after a few weeks—and radiation-induced pulmonary fibrosis that can develop several months or years after irradiation (IR)^4,5^. Modern clinical radiotherapy techniques, including stereotactic radiotherapy (SBRT)—which delivers small fractions of ablative radiation doses—are more accurate and precise than conventional radiotherapy techniques. Previously, we had developed a mouse model that simulates SBRT; we used an image-guided small-animal IR system to deliver a single high dose of radiation to the lung and verified the induction of pulmonary fibrosis in response to IR^6–8^.

Radiation pneumonitis develops within hours or days of lung IR and is accompanied by increased capillary permeability, leukocyte infiltration, and release of cytokines, such as transforming growth factor β (TGF-β), interleukin 6 (IL-6), tumor necrosis factor alpha, and interleukin-1 beta (IL-1β). Subsequent alveolar lung inflammation and persistent inflammation may lead to symptomatic phases, including pulmonary fibrosis^9,10^. TGF-β activation induces the differentiation of fibroblasts into myofibroblasts, expression of α-smooth muscle actin (α-SMA), and synthesis of extracellular matrix proteins, such as collagen^11,12^. Alveolar epithelial cells (AECs) are another important source of myofibroblasts and may differentiate into myofibroblasts during idiopathic pulmonary fibrosis and radiation-induced pulmonary fibrosis. This epithelial or endothelial–mesenchymal transition (EMT or EndMT) is generally known to be involved in the development of pulmonary fibrosis^13^. During EMT, cells undergo a morphological change from a round phenotype to a spindle-like phenotype, accompanied by a loss of epithelial cell markers—such as E-cadherin—and the gain of mesenchymal markers, such as vimentin and α-SMA^14^. In addition, NF-κB signaling—an important regulator of inflammatory responses—and TGF-β/Smad signaling are important regulatory mechanisms of EMT^12,13,15^.

The lipid mediator, LXA_4,_ is a bioactive product of arachidonic acid that has been reported to exert a variety of activities in multiple tissues, including anti-inflammatory effects, and regulation of neutrophil infiltration, pro-resolving signaling, macrophage polarization, and the nonphlogistic uptake of apoptotic polymorphonuclear neutrophils^16,17^. These activities of LXA_4_ are regulated by the G protein-coupled ALX/FPR2 receptor^18,19^. LXA4 reportedly reduces DM-related renal fibrosis by targeting TGF-β/Smad signaling and suppresses liver fibrosis in experimental models by regulating the immune responses and modulating the expression of regeneration genes^20,21^. It also lowers TGF-β levels in bleomycin-induced pulmonary fibrosis and exhibits an anti-fibrotic effect^22^. Furthermore, lipoxin inhibits EMT in cancer models, such as those of pancreatic and liver cancers, and LXA4 was found to inhibit EMT in a renal fibrosis model^23–25^. However, the role of lipoxin in RILI has not yet been reported.

Here, we examined the anti-inflammatory and anti-fibrotic properties of LXA_4_ using a mouse model of RILI, wherein ablative radiation doses were delivered to the focal lung area using an image-guided IR system^6,7,26–28^. We also investigated whether LXA_4_ regulates proteins that are important for EMT, particularly whether the crosstalk between LXA_4_-ALX/FPR2 and TGF-β/Smad signaling plays a key role in EMT.

## Results

### LXA_4_ inhibits radiation-induced lung inflammatory responses

**Fig. 1:**
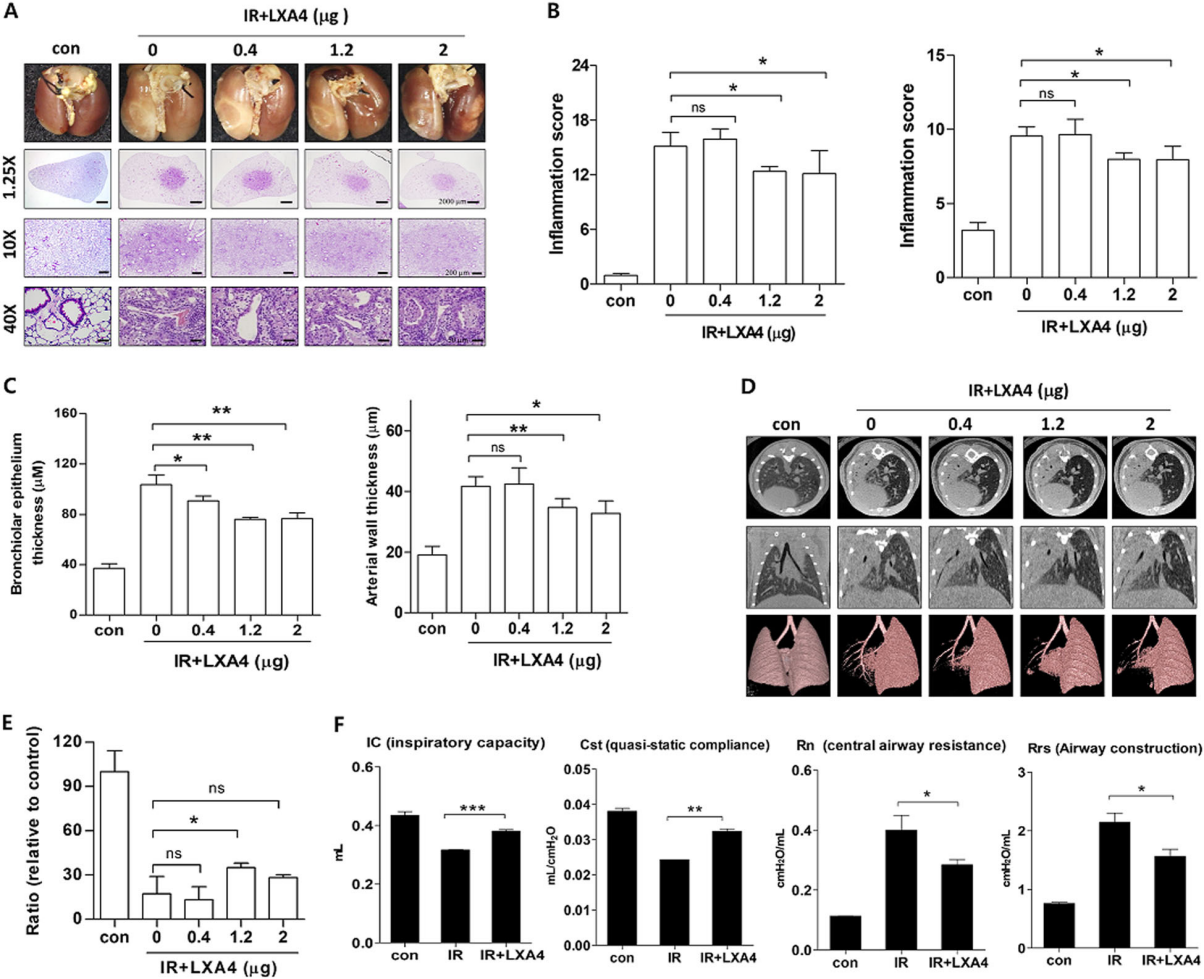
Effects of LXA_4_ on radiation-induced pneumonia 2 weeks after irradiation (IR). **a** Representative gross (top) and H&E (bottom) images of left lung tissues 2 weeks after 75 Gy IR with or without LXA_4_ in the mouse model. Magnification, ×1.25, ×10, and ×40. **b** Quantification of inflammatory foci at the lesion site (left) and in the surrounding lung tissue (right). **c** Quantification of bronchiolar epithelium (left) and arterial wall (right) thickness in lung tissues. **d** 3-D micro-computed tomography (CT) of mouse lung tissue. Horizontal (top), trans-axial (middle), and 3-D images (bottom) acquired 2 weeks post-IR. **e** Normal lung volume excluding consolidation was quantified using the micro-CT images. **f** Several mouse lung function parameters were measured using the flexiVent^TM^ system 2 weeks after irradiation, i.e., inspiratory capacity (IC), quasi-static compliance (Cst), central airway resistance (Rn), and airway construction (Rrs). Data are expressed as the mean ± standard error (*n* = 3, *p < 0.05, **p < 0.01, and ***p < 0.001).

To mimic the effects of clinical radiotherapy in an animal model of IR-induced lung damage, we had previously exposed mice lungs to high-dose radiation using a small-animal micro-irradiator (X-RAD320) equipped with a collimator system (to produce focal radiation beams)^7,8,28^. These studies showed that radiation pneumonitis occurs in mice within 2 weeks of exposure to 60‒100 Gy of IR^8,15,28^. Herein, we evaluated the effects of LXA_4_ on pneumonitis 2 weeks after 75 Gy IR by evaluating the lung morphology and performing hematoxylin and eosin (H&E) staining and pulmonary function assays (micro-computed tomography (CT) and flexiVent^TM^). The normal lung was brown in color, whereas the irradiated lung exhibited a ring-like boundary with white-colored adjacent areas 2 weeks post-IR (Fig. 1a, upper). The IR group displayed significantly more inflammation at the lesion site than the control group, and inflammation was significantly blocked by LXA_4_ (1.2 and 2 μg per mouse) in the IR + LXA_4_ group, which displayed less damage than the IR group (Fig. 1b, left). Inflammatory changes in the tissues surrounding the irradiated lesion were also significantly reduced in the LXA_4_-treated groups (1.2 and 2 μg/mouse) compared to those in the IR group (Fig. 1b, right). Further, LXA_4_ significantly reduced the IR-induced increases in the thickness of the bronchiolar epithelium and arterial wall (Fig. 1c). Micro-computed tomographic (micro-CT) images taken 2 weeks after IR show that pulmonary consolidation throughout the left lung of irradiated mice makes it possible to measure high air-tissue contrast^8,28^. We found that LXA_4_ reduced IR-induced pulmonary consolidation (Fig. 1d, e); therefore, we analyzed pulmonary function—based on six parameters—using the flexiVent^TM^ system (Supplementary Table 1). Four of these parameters showed significant improvement in the IR + LXA_4_ group, i.e., IC (inspiratory capacity; IR vs. IR + LXA_4_, 0.316 ± 0.0017 mL vs. 0.3812 ± 0.0044 mL), Cst (quasi-static compliance; IR vs. IR + LXA_4_, 0.02428 ± 0.00048 mL/cmH_2_O vs. 0.03231 ± 0.0006852 mL/cmH_2_O), Rn (airway construction; IR vs. IR + LXA_4_, 0.4000 ± 0.04930 cmH_2_O/mL vs. 0.2843 ± 0.01795 cmH_2_O/mL), and Rrs (central airway resistance; IR vs. IR + LXA_4_, 2.145 ± 0.1612 cmH_2_O/mL vs. 1.564 ± 0.1237 cmH_2_O/mL) (Fig. 1f).

**Fig. 2:**
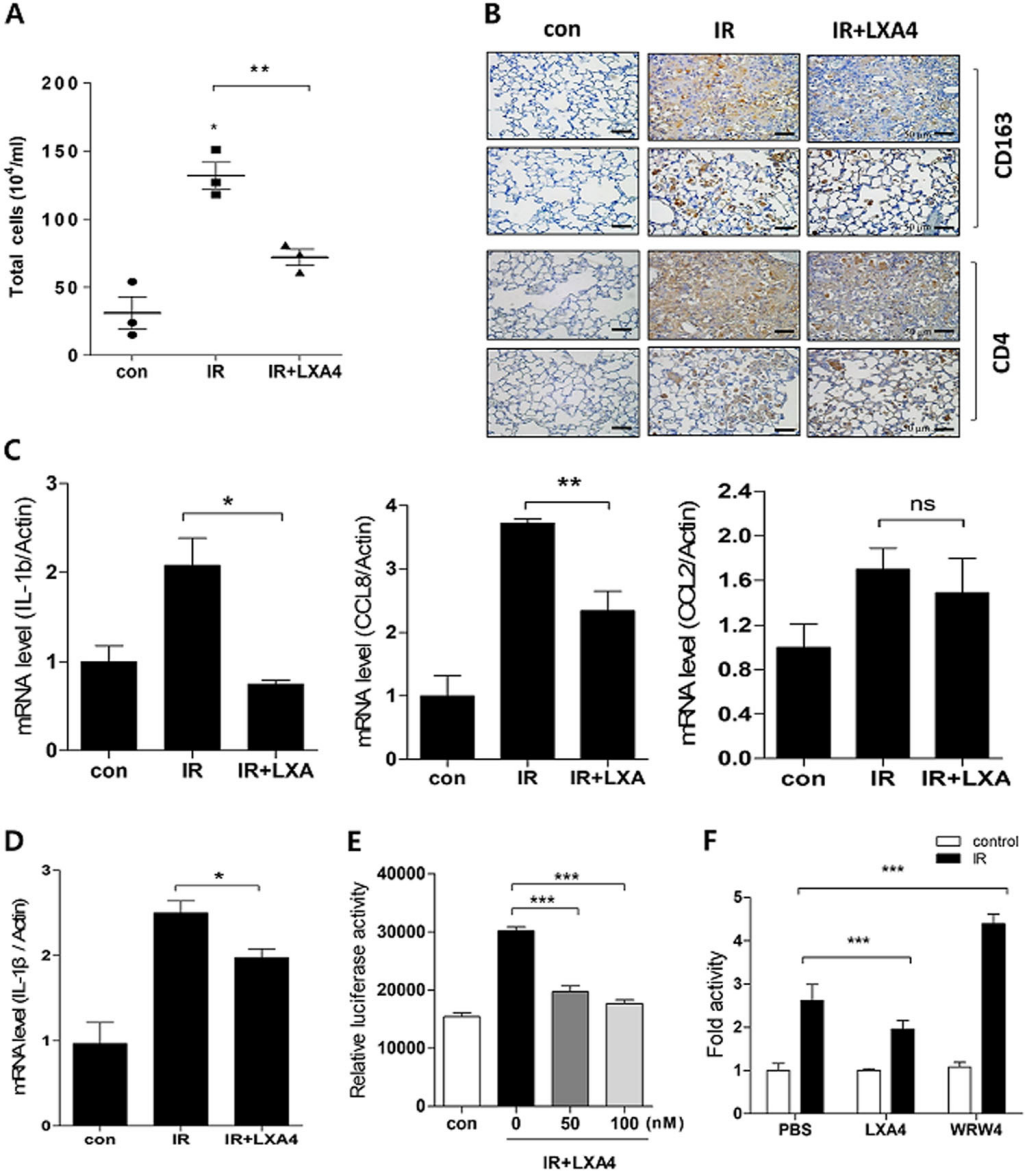
Effects of LXA_4_ on radiation-induced inflammatory response. **a** Total BALF-cell count in mice 2 weeks post-IR. **b** Representative immunohistochemical images of irradiated lung tissues using anti-CD163 and CD4 antibodies. Magnification, ×40. **c** IL-1b, CCL8, and CCL2 mRNA expression in irradiated lung tissue measured using quantitative real-time PCR. **d** IL-1b mRNA expression in MLE12 cells measured using quantitative real-time PCR. **e** pNF-κB reporter activity in MLE12 cells exposed to 6 Gy IR according to LXA4 dosage. **f** pNF-κB reporter fold activity in 6 Gy IR-exposed MLE12 cells transfected with or without WRW4. Data are expressed as the mean ± standard error of three independent experiments (*p < 0.05, **p < 0.01, and ***p < 0.001).

Bronchoalveolar lavage fluid (BALF) differential cell counts can provide supportive or even diagnostic information regarding various conditions, including lung inflammatory responses. IR increased the total number of BALF cells compared to that in the control group, whereas LXA_4_ rescued this effect and reduced the IR-induced infiltration of immune cells such as CD163+ and CD4+ cells (Fig. 2b). To confirm this anti-inflammatory effect, we evaluated inflammatory cytokine and chemokine expression, and found that while IR significantly upregulated IL-1b, CCL2, and CCL8 expression, LXA_4_ attenuated their expression in the mouse model (Fig. 2c). Similarly, LXA_4_ decreased the IR-induced increase in IL-1b expression in MLE12 cells (Fig. 2d), and significantly attenuated IR-induced NF-κB promoter activity—which mediates inflammatory gene transcription—in a dose-dependent manner (Fig. 2e). However, WRW4—a specific FPR2 antagonist—blocked the effect of endogenous LXA4 and enhanced IR-induced NF-κB promoter activity (Fig. 2f).

### LXA_4_ attenuates radiation-induced pulmonary fibrosis

**Fig. 3:**
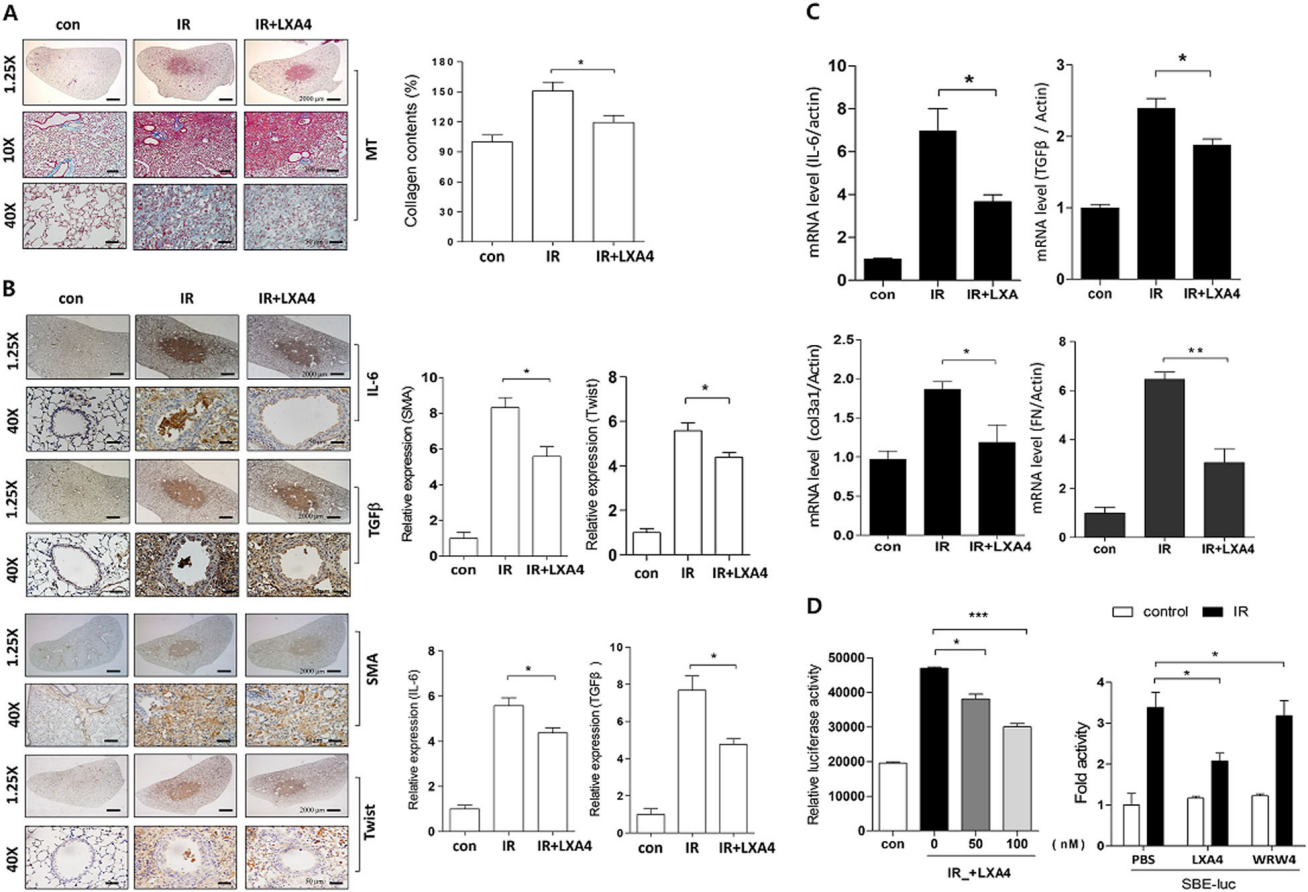
LXA_4_ inhibits IR-induced pulmonary fibrosis at 2 weeks. **a** Representative MT-stained image of mouse lung tissue 2 weeks post-IR (75 Gy) with or without LXA_4_ in the mouse model (left). Quantification of collagen content (right). Magnification, ×1.25, ×10, and ×40. **b** Representative immunohistochemical image (left) and quantification (right) of irradiated lung tissues stained with antibodies against IL-6, TGF-β, α-SMA, and Twist. Magnification, ×1.25 and ×40. **c** IL-6, TGF-β, Col3al, and fibronectin (FN) mRNA expression in the irradiated lung tissue of mice, measured using quantitative real-time PCR. **d** pSmad reporter activity in 6 Gy IR-exposed MLE12 cells transfected with or without WRW4. Data are expressed as the mean ± standard error of three to five independent experiments (*p < 0.05, **p < 0.01, and ***p < 0.001).

Previously, we had observed that the inflammatory response occurred for around 2 weeks, and that fibrosis was apparent 4‒6 weeks after ablative doses of IR^6,8,28^. Interestingly, we began to observe collagen deposition—using Masson’s trichrome (MT) staining—2 weeks after IR, with collagen deposition increasing thereafter (Fig. 3a). This early fibrosis was attenuated by LXA_4_ as was the expression of proteins involved in pulmonary fibrosis, including IL-6, TGF-β, α-SMA, and Twist (Fig. 3b). Moreover, LXA_4_ decreased IL-6, TGF-β, Col3a1, and fibronectin (FN) mRNA levels, which were increased by IR in MLE12 cells (Fig. 3c). Next, we performed a luciferase reporter assay to determine whether the effect of FPR2 on Smad-binding element (SBE) activity mediated TGF-β/Smad signaling. IR increased SBE activity, whereas LXA_4_ treatment significantly attenuated this increase (Fig. 3d, left). However, WRW4, a specific FPR2 antagonist, blocked the effect of endogenous LXA4 and increased IR-induced SBE-luciferase promoter activity (Fig. 3d, right). Thus, LXA_4_ may regulate collagen deposition and protein expression—associated with early RILI—and inhibit inflammation after IR.

**Fig. 4:**
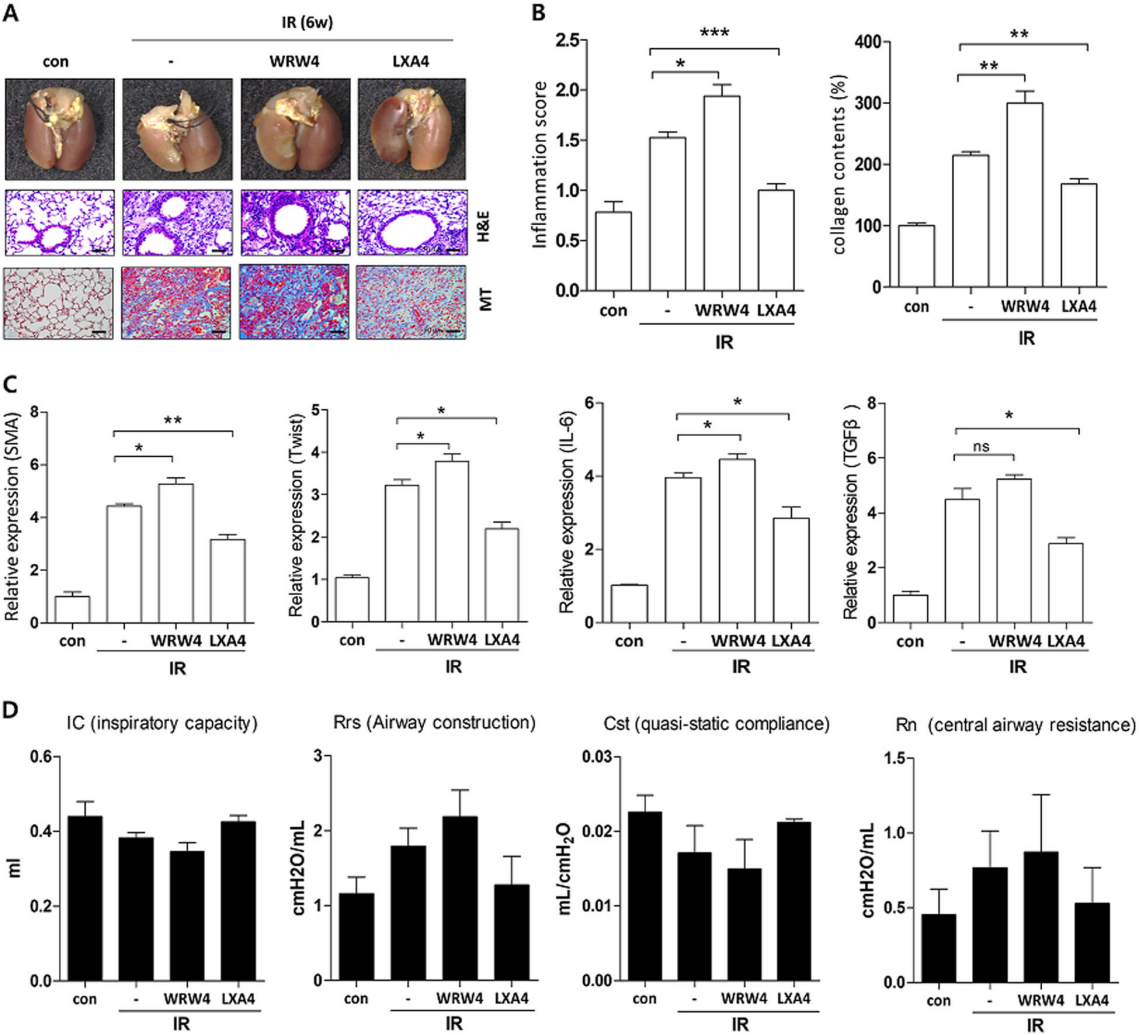
LXA_4_ attenuates IR-induced pulmonary fibrosis and rescues lung function. **a** Representative gross (top), H&E (middle), and MT (bottom) images of the left lung tissue 6 weeks after 75 Gy IR with or without LXA4 in the mouse model. Magnification, ×40. **b** Quantification of inflammatory foci (left) and collagen content (right). **c** Quantification of relative α-SMA, Twist, IL-6, and TGF-β expression in the lung lesion site using immunohistochemistry. **d** Functional measurement of several mouse lung parameters using the flexiVent^TM^ system 6 weeks post-IR, i.e., inspiratory capacity (IC), quasi-static compliance (Cst), central airway resistance (Rn), and airway construction (Rrs). Data are expressed as the mean ± standard error (*n* = 3‒5, *p < 0.05, **p < 0.01, and ***p < 0.001).

Six weeks after IR, LXA_4_ decreased the radiation-induced increase in immune-cell infiltration and collagen expression to match the levels in the early stage IR (Fig. 4a, b). Furthermore, we confirmed that WRW4, an FPR2 antagonist that blocks the effect of endogenous LXA_4_, was upregulated in the IR-treated group (Fig. 4a, b). Bronchiolar epithelium thickness and arterial wall thickness also responded to the inhibitory effects of LXA_4_ and WRW4 (data not shown). Moreover, the expression of α-SMA, Twist, IL-6, and TGF-β was significantly inhibited by LXA_4_ and increased by WRW4 (Fig. 4c). Lung function was rescued by LXA_4_, which significantly reversed four of the six lung function parameters (IC, Cst, Rrs, and Rn), and was worsened by WRW4, but this was not significant (Fig. 4d). These results demonstrate that LXA_4_ may inhibit the development of radiation-induced fibrosis via the FPR2 receptor.

### Radiation affects LXA4-FPR2 signaling

**Fig. 5:**
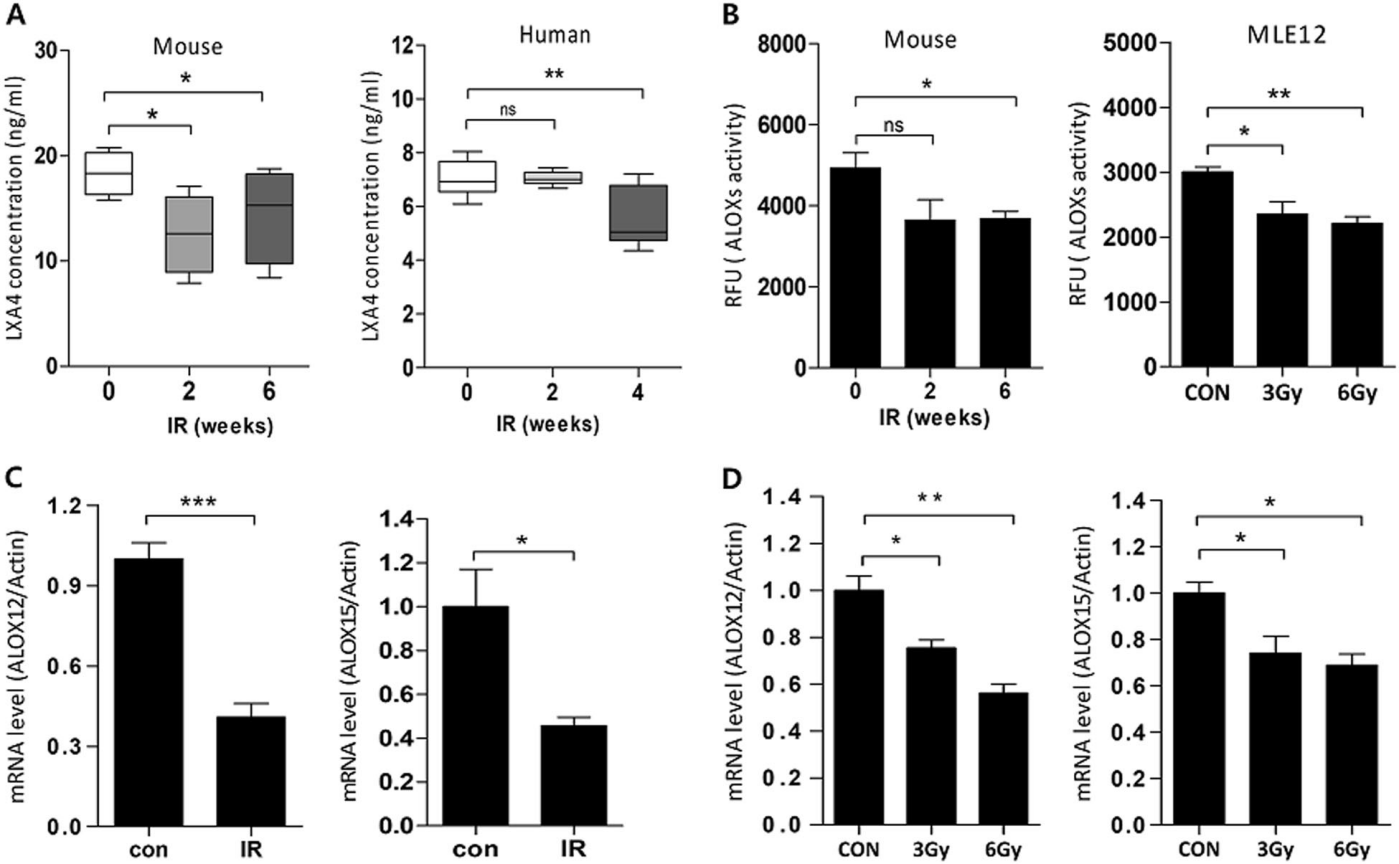
IR reduces LXA_4_ production. **a** Determination of plasma LXA_4_ concentration in mice. Plasma samples were collected from mice 2 and 6 weeks post-75 Gy IR and their LXA_4_ levels were measured by ELISA. **b** LOX enzyme activity was measured in mice (left) and MLE12 cells (right) using a Lipoxygenase Assay kit, according to the manufacturer’s instructions. ALOX12 and ALOX15 mRNA levels in the irradiated lung tissues of mice (**c**) and MLE12 cells (**d**) measured using quantitative real-time PCR. Data are expressed as the mean ± standard error (*n* = 3‒5, *p < 0.05, **p < 0.01, and ***p < 0.001).

Next, we explored whether IR affects LXA_4_ levels using enzyme-linked immunosorbent assays (ELISA) on mouse blood samples. As shown in Fig. 5a (left), endogenous LXA_4_ levels were significantly lower 2 (12.56 ± 1.885 ng/mL) and 6 (14.46 ± 2.248 ng/mL) weeks after IR than those in the control group (18.33 ± 1.037 ng/mL). Similarly, LXA_4_ levels in the human blood (Fig. 5a, right panel) were significantly lower after 4 weeks of IR (5.527 ± 0.4614 ng/mL) than those in the control group (7.044 ± 0.2798 ng/mL). IR also reduced the lipoxygenase activity in the mouse model and MLE12 cells (Fig. 5b) and downregulated Alox12 (12-lipoxygenase) and Alox15 (15-lipoxygenase), lipoxygenase variants—involved in arachidonic acid metabolism—in mouse lung lesion sites (Fig. 5c) and MLE12 cells (Fig. 5d).

**Fig. 6:**
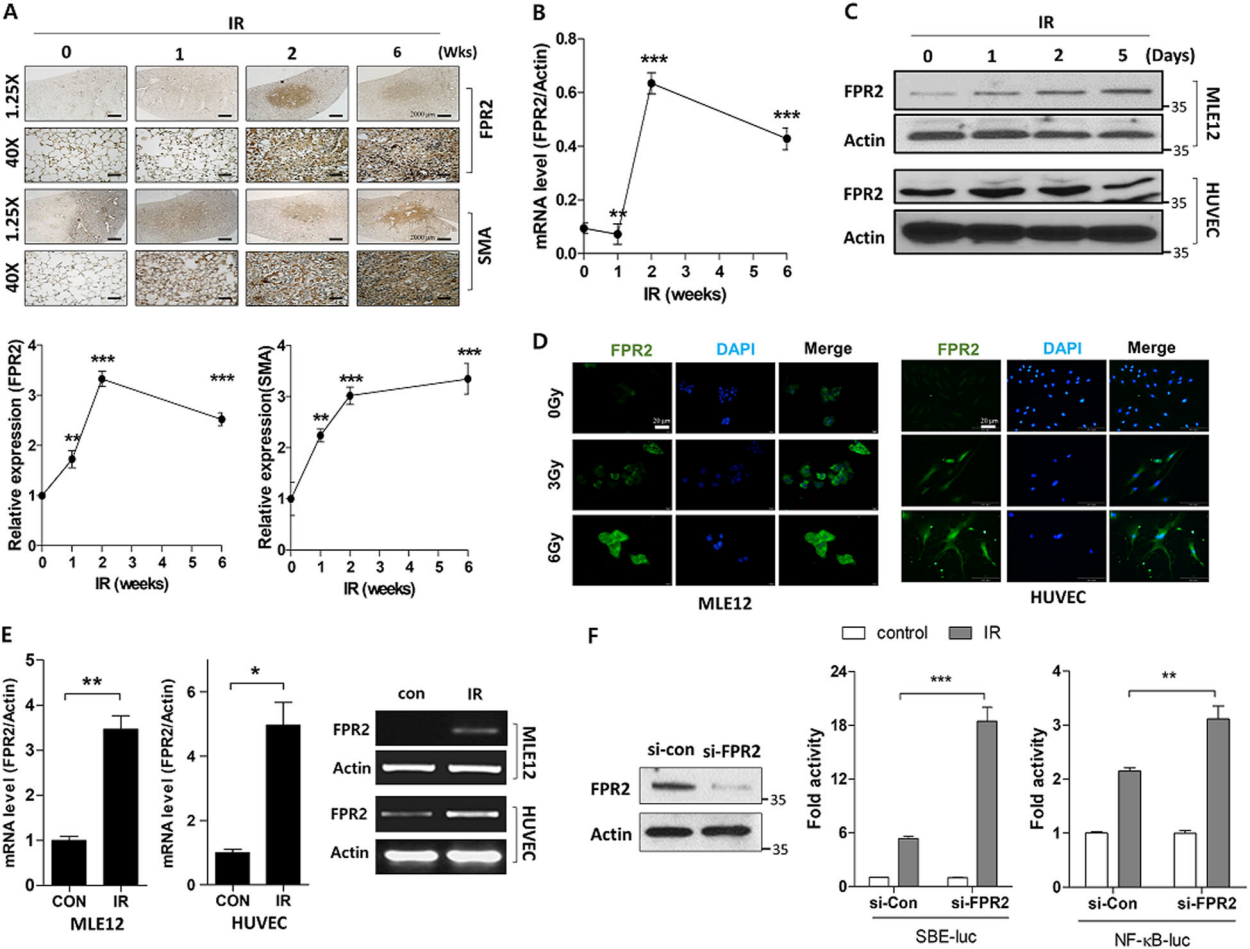
IR regulates ALX/FPR2 expression. **a** Representative image (top) and quantification (bottom) of irradiated lung tissues (75 Gy) stained for FPR2 and α-SMA at the indicated times. Magnification, ×1.25 and ×40. **b** FPR2 mRNA levels in IR-exposed mouse lung tissue measured using quantitative real-time PCR. **c** FPR2 expression measured by western blotting after exposure of MLE12 cells and HUVECs to 6 Gy IR. **d** FPR2 expression 24 h after 3 and 6 Gy IR in MLE12 cells and HUVECs; FPR2 (green); DAPI (blue). **e** FPR2 mRNA levels in MLE12 cells and HUVECs exposed to 6 Gy IR, measured using quantitative real-time PCR. **f** pSmad and pNF-κB promoter reporter activity in 6 Gy IR MLE12 cells transfected with or without FPR2 siRNA. Data are expressed as the mean ± standard error (*n* = 3‒5, *p < 0.05, **p < 0.01, and ***p < 0.001).

To investigate FPR2 expression, we focally irradiated the left lung of mice (3-mm diameter) with high-dose radiation (75 Gy) at the indicated times. IR increased the degree of pulmonary damage, as evidenced by inflammatory-cell infiltration—H&E staining—and collagen content—MT staining—in the IR-induced lung damage model in mice (Supplementary Fig. 1). Moreover, α-SMA expression—related to fibrosis—increased in the irradiated left lung over time. Interestingly, IR significantly increased FPR2 levels at the lesion site (Fig. 6a, 75 Gy; and Supplementary Fig. 2, 90 Gy) alongside FPR2 mRNA levels 2 weeks after IR; however, FPR2 mRNA levels were lower 6 weeks after IR (Fig. 6b). We confirmed IR-induced FPR2 expression in the mouse lung epithelial cell line—MLE12—and in human endothelial cells (HUVECs) using western blotting (Fig. 6c), immunofluorescence (Fig. 6d), and real-time quantitative RT-PCR (Fig. 6e); these showed that FPR2 levels significantly increased after 48 h of IR. Next, we performed a luciferase reporter assay to determine the effect of FPR2 on SBE and NF-κB promoter activities, which regulate TGF-β signaling and inflammatory responses. Interestingly, IR increased the activities of both promoters in MLE12 cells—as did si-FPR2—compared to those in control (Fig. 6f). Therefore, these data suggest that the altered LXA_4_ signaling, including increased FPR2 expression and crosstalk with TGF-β/Smad signaling, may be involved in the development of IR-induced pulmonary damage.

### LXA_4_ blocks IR-induced cell death and oxidative response

**Fig. 7:**
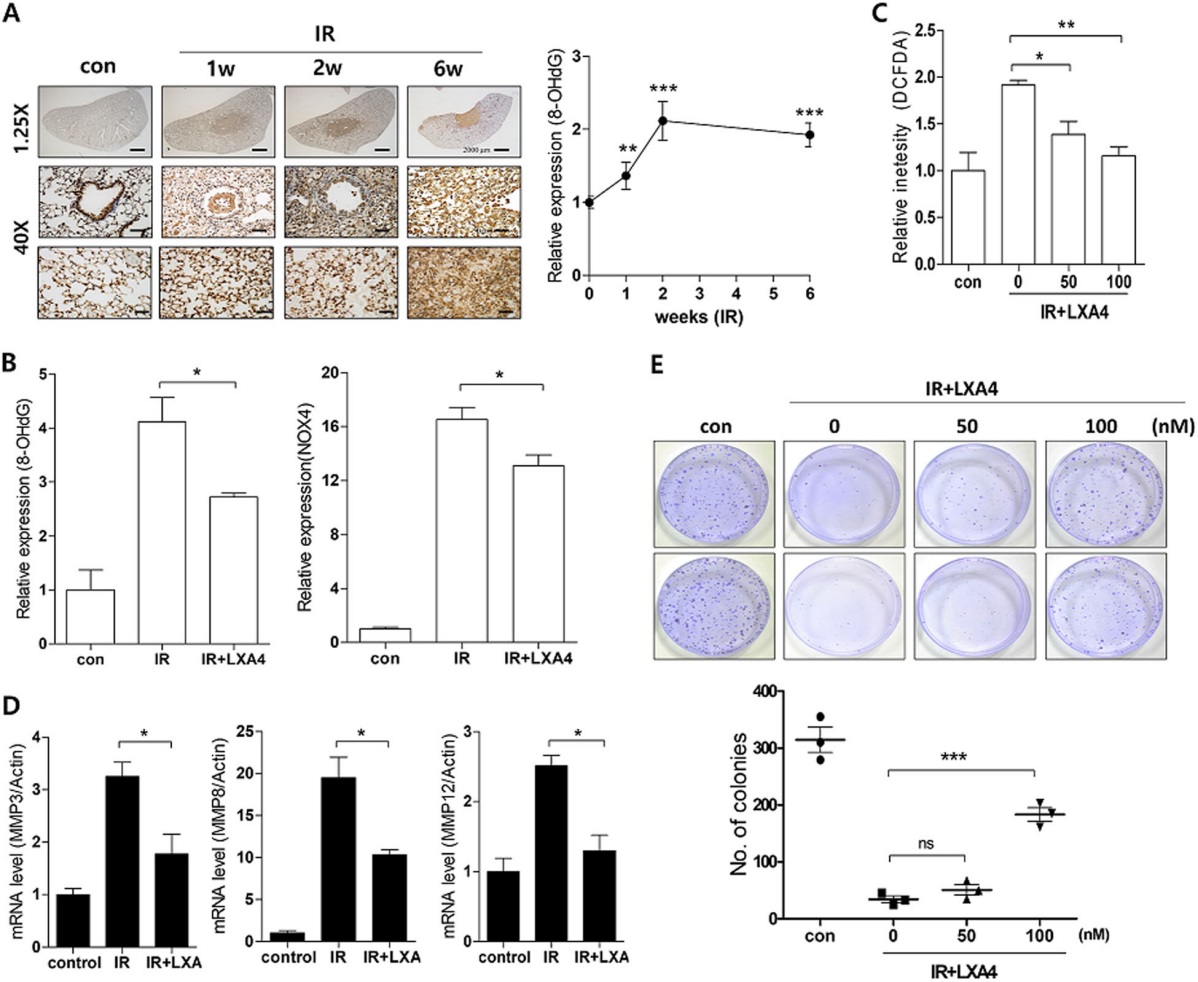
LXA_4_ decreases the IR-induced oxidative response. **a** Representative image (right) and quantification (left) of irradiated lung tissues stained with antibodies against 8-OHdG at the indicated times. Magnification, ×1.25 and ×40. **b** Quantification of relative 8-OHdG and NOX4 expression in the lung lesion site 6 weeks after 75 Gy IR with or without LXA_4_ using immunohistochemistry. **c** ROS levels were measured by incubating irradiated MLE12 cells treated with or without LXA_4_ with a DCFDA probe. **d** MMP3, MMP8, and MMP12 mRNA expression in IR-exposed mouse lung tissue, measured using quantitative real-time PCR. **e** Colony forming activity was examined in MLE12 cells. Data are expressed as the mean ± standard error (*n* = 3, *p < 0.05, **p < 0.01, and ***p < 0.001).

IR increased the expression of the critical free radical marker, 8-hydroxy-2′-deoxyguanosine (8-OHdG) over time (Fig. 7a); therefore, we examined 8-OHdG levels and NOX4 expression—which is involved in reactive oxygen species (ROS) modulation—using immunohistochemistry in irradiated mouse lung models to observe the effect of LXA_4_ on radiation-induced oxidative stress. 8-OHdG and NOX4 levels were increased 6 weeks post-IR, and were significantly decreased by LXA_4_ (Fig. 7b). Moreover, LXA_4_ reduced IR-induced ROS production in MLE12 cells—observed using a DCFDA probe (Fig. 7c)—and also reduced MMP expression (Fig. 7d) and cell death (Fig. 7e). These data suggest that LXA_4_ can block IR-induced increase in cell death and oxidative response.

## Discussion

As the use of radiation for treating cancer—and the number of patients surviving cancer—is increasing worldwide, it has become increasingly important to improve the quality of life of these individuals and to modulate the effects of radiation therapy on normal tissues^29^. IR increases inflammatory reactions, thereby causing inflammatory cells to accumulate in the lesion sites of lung tissue, enhancing inflammatory cytokine expression (IL-6, IL-1b, and TGF-β) in lung tissues, and recruiting immune cells (macrophages, neutrophils, lymphocytes, and eosinophils) in BALF^6,28^. To our knowledge, this study is the first to demonstrate the effect of lipoxin on RILI; it reveals that LXA_4_ exhibits anti-inflammatory and anti-fibrotic effects in a RILI model. LXA_4_ not only inhibited radiation-induced pro-inflammatory cytokine production but also suppressed immune-cell recruitment into the alveolar space, downregulated fibrosis-related gene expression, and restored the damaged lung tissue and function, thereby ameliorating RILI.

Although lipoxins were discovered almost four decades ago, they have not yet reached the clinical trials stage—as potential drugs to treat inflammatory diseases—owing to their short half-life and instability on exposure to acids and light; however, stable analogs are being developed. In this study, we predominantly investigated the anti-fibrotic effects of LXA_4_—2 and 6 weeks after 75 Gy of IR—by immunostaining for α-SMA, TGF-β, and Twist and by MT staining for collagen expression. Two weeks after exposure to 75 Gy IR, our experimental model manifested radiation pneumonitis with radiation-induced inflammation, whereas 6 weeks after 75 Gy IR exposure, the model manifested IR-induced lung fibrosis with increased fibrosis marker and collagen expression^8,26,28^. To investigate the crosstalk between FPR2, IR-induced inflammatory responses, and fibrosis-related gene expression, we observed NF-κB and SBE promoter activity in the background of IR. NF-κB regulates inflammation-related signals, and its activity is known to increase during various inflammatory reactions, including radiation. We confirmed that IR increased NF-κB and SBE promoter activities in TGF-β/Smad signaling, which regulates the expression of fibrotic genes. In addition, administering si-FPR2 and WRW4 to reduce the effects of endogenous FPR2 further increased the activities of these two promoters in the IR group, whereas LXA_4_ suppressed their activities. To investigate the effects of LXA_4_ against IR-induced fibrosis, we performed a variety of assays. Immunohistochemistry for 8-OHdG and NOX4 revealed that LXA_4_ inhibited oxidative stress, suggesting that LXA_4_ may have antioxidant properties. IR-mediated ROS are known to induce apoptosis in lung epithelial cells^30^, which can cause pulmonary fibrosis;^31^ although inhibition of AEC apoptosis has been shown to attenuate fibrosis^32^, it did not affect IR-induced cancer cell death in A549 cells (Supplementary Fig. 4).

Chest CT and studies on pulmonary function are important factors to consider when clinically evaluating respiratory diseases. micro-CT is often used in preclinical studies to measure lung injury as it allow the therapeutic effects of drugs to be evaluated quickly and noninvasively. FlexiVent^TM^ is a system—with a pre-programmed ventilator—that directly evaluates lung function based on the functional parameters used in humans^33^. Here, LXA_4_-treated mice displayed significantly better micro-CT finding and flexiVent^TM^ lung function values than simply irradiated mice. Moreover, these results correlated with the histopathological findings—H&E and MT staining—suggesting that LXA_4_ can prevent IR-induced lung function deterioration.

LXA_4_ is reportedly involved in airway inflammation; its levels decrease in chronic airway inflammatory diseases, such as asthma, chronic obstructive pulmonary disease, and cystic fibrosis^34,35^. Moreover, a reduced proportion of pro-resolving compounds (LXA_4_)—compared to pro-inflammatory compounds (LTB_4_)—is associated with decreased lung function^36^. LXA_4_ is generated during the interaction of lipoxygenases (LOXs) via trans-cellular cooperation in several immune cells, including neutrophils, eosinophils, alveolar macrophages, and platelet airway epithelial cells^37–39^. Here, IR decreased LXA_4_ production—in the mouse model and in human blood samples—LOX activity and expression of ALOX12 and ALOX15. Conversely, IR increased the expression of the LXA_4_ receptor FPR2 in the mouse lung tissue model and in MLE12 cells and HUVECs. Further studies should investigate FPR2-LXA_4_ interactions in lung cells at different time points after IR, in addition to investigating the effect of LXA_4_ on aspects of radiation-induced inflammation, such as inflammasome activation and immune-cell infiltration, which promote immune resolution.

Consistent with our findings, LXA_4_ signaling has recently emerged as an endogenous anti-inflammation and anti-fibrosis pathway^40–42^; however, natural LXA_4_ has low biological efficacy owing to its instability. To overcome these limitations, many researchers have developed analogs—that mimic the core structure of native LXA_4_—as potential drugs^43,22^. Therefore, it may be beneficial to introduce and develop these analogs in our radiation-induced lung damage model to maximize the anti-inflammation and anti-fibrotic effects of LXA_4_.

In conclusion, although the precise mechanisms underlying radiation-induced lung fibrosis remain unclear, we demonstrated that LXA_4_ exerts protective effects by inhibiting collagen production, decreasing the expression of fibrosis-related proteins and inflammatory cytokines, and blocking NF-κB and TGF-β/Smad signaling. Collectively, these results suggest that LXA_4_ reduces radiation-induced lung inflammation and fibrosis and may therefore serve as a useful therapeutic agent for inhibiting radiotherapy-induced inflammatory responses and fibrosis in normal lung tissue.

## Materials and methods

### Animal experiments and LXA_4_ administration

All protocols involving the use of mice were approved by the Animal Care and Use Committees of Yonsei University Medical School, Seoul, South Korea (YUHS-IACUC; 2016-0199), and were performed in accordance with the relevant guidelines. Male C57BL/6 mice (age, 6 weeks; weight, 20–25 g) were purchased from Charles River Korea (Orient Bio, Seongnam, South Korea) and allowed to acclimatize (*n* = 5 per cage) for a week before IR. A single dose of 75 Gy IR was delivered to the left lung using an image-guided small-animal irradiator (X-RAD 320; Precision, North Branford, CT, USA) equipped with a collimator comprising 3.5-cm-thick copper—for producing focal radiation beams—and an imaging subsystem comprising a fluorescent screen coupled to a charge-coupled-device camera. In all experiments, 3-mm collimators were used to mimic clinical SBRT conditions with a small IR volume in lung tissues. The mice were randomly divided into the following three groups (*n* = 3‒5 per group): (1) control group; (2) IR group exposed to a single dose of 75 Gy delivered to the left lung in a single fraction; (3) IR + LXA_4_ group treated intravenously with LXA_4_ (0.4, 1.2, or 2 µg/ mouse) 2 weeks after IR. The mice were euthanized by CO_2_ inhalation, and their lung tissues were collected for analysis. LXA_4_ levels were measured in serum samples using an LXA_4_ ELISA kit (Cusabio Biotech, Wuhan, China) according to the manufacturer’s protocol.

### Human blood specimens

The collection of blood samples from patients with lung cancer was approved by the Ethics Committee of Severance Hospital, Yonsei University, Seoul, South Korea (4-2014-0193). After informed consent, 10-mL blood samples were collected from each patient before radiotherapy to determine LXA_4_ levels in the serum, at the 2nd and 4th week after definitive radiotherapy for curative aim. The samples were centrifuged, and the serum was frozen and stored at −70 °C until further analysis. LXA_4_ levels were measured in serum samples using an LXA_4_ ELISA kit, as described above.

### Cell culture

HUVECs were kindly provided by Dr. Yoon-Jin Lee (Division of Radiation Effects, Korea Institute of Radiological and Medical Sciences, Seoul, Korea) and were grown in Endothelial Growth Medium-2 (EGM-2; Promocell GmbH, Heidelberg, Germany) in 0.1% gelatin-coated dishes at 37 °C in a humidified atmosphere containing 5% (v/v) CO_2_. The mouse lung epithelial cell line, MLE12, obtained from the American Type Culture Collection, was grown in Dulbecco’s modified Eagle’s medium (DMEM) supplemented with 10% fetal bovine serum (FBS), 100 U/mL penicillin, and 100 mg/mL streptomycin at 37 °C in a humidified atmosphere containing 5% (v/v) CO_2_. The cells were seeded (density, 1.0 × 10^6^ cells) in a 60-mm plate. After 24 h, the cells were washed and maintained in serum-free medium before the experiments.

### Preparation and histological evaluation of lung tissue sections

Tissues from the left lung of the irradiated mice were fixed in 4% paraformaldehyde for 24 h, dehydrated, and embedded in paraffin. Tissue sections were stained with H&E, MT, and TGF-β, α-SMA, 8-OHdG, and IL-6 antibodies (Abcam, Cambridge, MA, USA).

### Immunohistochemistry and immunofluorescence

To detect TGF-β, α-SMA, 8-OHdG, and IL-6, tissue sections were probed with primary antibodies (4 °C overnight), followed by incubation with the avidin–biotin peroxidase complex (ABC Kit, Vector Laboratories, CA, USA), and were developed using 3, 3′-diaminobenzidine tetrachloride (DAB; Zymed Laboratories, CA, USA). For immunofluorescence, tissues were probed overnight with primary antibodies diluted in phosphate buffered saline (PBS) containing 0.5% bovine serum albumin (BSA) and then incubated overnight with fluorescent-conjugated secondary antibodies (Vector Laboratories, CA, USA)—diluted in the same buffer as the primary antibodies—at 4 °C. These sections were mounted on a gelatin-coated slide using a fluorescent mounting medium (Dako, Glostrup, Denmark) and dried.

### Immunocytochemistry

Cells were fixed with 4% paraformaldehyde—in PBS—permeabilized with 0.1% Triton X-100 for 10 min at room temperature, and washed several times with PBS. The cells were then incubated with pSmad2/3 primary antibodies (Abcam, Cambridge, MA, USA) at 4 °C overnight, blocked with 1% BSA in PBS, and stained with secondary Texas Red-conjugated antibodies. Next, the cells were stained with DAPI, mounted on coverslips, and observed under a confocal microscope.

### Western blotting

Cells were lysed with RIPA buffer (50 mM Tris-HCl, pH 7.4; 1% Nonidet P-40; 0.25% sodium deoxycholate; 150 mM NaCl; 1 mM Na_3_VO_4_) containing protease inhibitors (2 mM phenylmethylsulfonyl fluoride; 100 μg/mL leupeptin; 10 μg/mL pepstatin, 1 μg/mL aprotinin; 2 mM EDTA) and a phosphatase inhibitor cocktail (GenDEPOT, Baker, TX, USA). After incubation for 20 min at 4 °C, the lysates were centrifuged at 13,000 rpm for 20 min at 4 °C, and the supernatants were collected for western blotting. Protein concentration was measured using a BCA protein kit (Bio-Rad, Hercules, USA). The lysates were subjected to SDS-PAGE gel electrophoresis, transferred onto polyvinylidene fluoride membranes (Millipore, Bedford, MA, USA), and incubated with 5% skimmed milk for 1 h at room temperature. The membranes were then probed with primary antibodies against α-SMA (Abcam, Cambridge, MA, USA), FPR2 (Novus Biological, Littleton, CO, USA), and actin (Santa Cruz Biotechnology, Dallas, TX, USA), followed by incubation with horseradish peroxidase-coupled secondary antibodies (Jackson Immunoresearch, West Grove, PA, USA). Proteins were detected using ECL Plus western blotting detection reagents (Amersham Biosciences, Piscataway, NJ, USA).

### micro-CT analysis

Micro-CT images were acquired using a volumetric CT scanner (NFR-Polaris-G90MVC: NanoFocusRay, Iksan, South Korea) and reconstructed using volumetric cone-beam reconstruction (Feldkamp–Davis–Kress method) in online/offline modes. Volumetric analysis was performed using ImageJ software (http://imagej.nih.gov/ij/). To minimize inter-specimen measurement variation, the same settings were used to analyze all images.

### Functional lung assessment

Lung function in irradiated mice was evaluated using a flexiVent^TM^ system (SCIREQ, Montreal, QC, Canada), which measures flow-volume relationships in the respiratory system—including forced oscillation—to discriminate between airway and lung tissue variables^27^. This system was used according to the manufacturer’s instructions.

### Transfection and reporter gene assay

Transient transfection was performed using Effectene (Qiagen; Santa Clara, CA, USA) according to the manufacturer’s protocol. Briefly, 1.0 × 10^6^ cells were seeded in a 60-mm dish a day before transfection and grown to ~70% confluence. The cells were then transfected with 0.1 µg of plasmid DNA—SBE-luc from the SBE Reporter Kit (BPS Bioscience, CA, USA)—or the NF-κB-luc vector pGL4.32 [luc2P/ NF-κB-RE/Hygro] (Promega, Madison, WI, USA) along with 10 nM siRNA against FPR2 (si-FPR2) or control siRNA. The next day, the transfected cells were treated with or without radiation (6 Gy) for 48 h and harvested for protein extraction, real-time-qPCR, or further analysis. All experiments were performed in triplicate, and representative results were reported. For the reporter assay, cells were lysed and assayed for luciferase activity according to the manufacturer’s instructions (Promega, Southampton, UK).

### Quantitative real-time polymerase chain reaction (qRT-PCR)

Total RNA was isolated from cells using TRIzol (Invitrogen, Carlsbad, CA, USA) according to the manufacturer’s instructions, and cDNA was synthesized using a Quantifect Reverse Transcription Kit (Qiagen, Hilden, Germany). qRT-PCR was performed using SYBR Premix Ex Taq (Takara, Hercules, CA, USA), primers, RNase-free H_2_O, and cDNA (final reaction volume, 20 µL) under the following cycling conditions: 95 °C (10 min); and 50 cycles of 95 °C, (20 s), 55 °C (30 s), and 72 °C (20 s). The mouse primer sequences are available in the Supplementary Table 2. All experiments were performed in triplicate, and the results were normalized to glyceraldehyde 3-phosphate dehydrogenase expression. mRNA expression was calculated using the delta Ct method.

### Luciferase reporter assay

MLE12 cells were cultured in 60-mm plates using complete medium until they reached ~70% confluency, and were then co-transfected with either SBE-luciferase + TK-*Renilla* luciferase or NF-κB-luciferase + TK-*Renilla* luciferase. After 24 h, the cells were irradiated using an image-guided small-animal irradiator, and the ratio of firefly/*Renilla* luciferase was calculated and normalized to untreated controls. Luciferase activity was measured using luciferase assay reagents (Promega Corp., Madison, Wi, USA).

### Colony formation assay

A549 and MLE12 cells were cultured in 35-mm dishes using DMEM supplemented with 10% FBS in the background of IR (5 Gy) using an X-rad320 (Precision, North Branford, CT, USA). After 2 weeks—with or without LXA4 treatment—the cells were fixed, stained with 0.5% crystal violet, and quantified for colony number.

### Statistical analysis

Statistical analysis was performed using GraphPad Prism 5 (GraphPad Software, La Jolla, CA, USA). Differences between the means of two groups were evaluated using Student’s *t* tests. Differences between the means of multiple groups were evaluated by one-way analysis of variance. The threshold for significance was *p* < 0.05, and all values were expressed as the mean ± SEM.

## Supplementary information

Supplementary information

Supplementary figure legends

Supplementary table 1 & 2

Supplementary figure 1

Supplementary figure 2

Supplementary figure 3

Supplementary figure 4
