# Supplementary material for: LXA4-FPR2 signaling regulates radiation-induced pulmonary fibrosis via crosstalk with TGF-β/Smad signaling
Source: Cell Death Dis. 2020 Aug 8;11(8):653. doi: 10.1038/s41419-020-02846-7 (PMC7434774; doi:10.1038/s41419-020-02846-7)
Supplement: Supplementary file 2 — Supplementary figure legends [file 41419_2020_2846_MOESM2_ESM.docx]

**Supplementary figure legends**

**Supplementary figure** 1. Representative image (top) and quantification (bottom) of H&E and MT staining of irradiated (90 Gy) mice lung tissues of at indicated times**.** Magnification, 12.5´ and 10´. Data are expressed as the mean ± standard error (**p* < 0.05, ***p* < 0.01, and ****p* < 0.001).

**Supplementary figure 2.** Representative image (top) and quantification (bottom) of immunohistochemical staining of irradiated (90 Gy) mice lung tissues at indicated times, which were stained for FPR2. Magnification, 12.5´ and 40´. Data are expressed as the mean ± standard error (**p* < 0.05, ***p* < 0.01, and ****p* < 0.001).

**Supplementary figure 3.** IR increases the expression of FPR2 in lesion sites of mouse lung. The expression of FPR2 (red) was co-stained with SMA (green) using immunofluorescence staining in irradiated mice lung tissue.

**Supplementary figure 4.** LXA4 does not affect IR-induced tumor cell death. Colony forming activity was examined in A549. Data are expressed as the mean ± standard error (n=3, ns; no significance)

**Supplementary table. 1.** The parameters of flexiVent^TM^ assay.

**Supplementary table 2.** Primer sequence
