# Supplementary material for: LXA4-FPR2 signaling regulates radiation-induced pulmonary fibrosis via crosstalk with TGF-β/Smad signaling
Source: Cell Death Dis. 2020 Aug 8;11(8):653. doi: 10.1038/s41419-020-02846-7 (PMC7434774; doi:10.1038/s41419-020-02846-7)
Supplement: Supplementary file 3 — Supplementary table 1 & 2 [file 41419_2020_2846_MOESM3_ESM.docx]

**Supplementary table 1. The parameters of flexiVent^TM^ assay**

| Parameter | Abbreviations | Description |
| --- | --- | --- |
| Inspiratory Capacity | IC | IC is the volume difference between functional residual capacity (FRC) and total lung capacity (TLC), also equaling tidal volume plus the inspiratory reserve volume. |
| Quasi-static Compliance | Cst | The parameter K of the Salazar-Knowles equation reflects the curvature of the upperportion |
| Tissue Damping | G | Tissue damping is closely related to tissue resistance and reflects the energy dissipationin the lung tissues. |
| Tissue Elastance | H | The parameter H is closely related to tissue elastance and reflects the energy conservation in the lung tissues. |
| Newtonian Resistance | Rn | The Newtonian Resistance parameter of the Constant Phase Model represents the resistance of the central airway |
| Airway constriction | Rrs | Resistance of the respiratory system |

**Supplementary table 2. Primer sequence**

|  | **Forward sequence** |  | **Reverse sequences** |
| --- | --- | --- | --- |
| **IL-1beta** | 5ʹ -TGG TGT GTG ACG TTC CCA TT-3ʹ |  | 5ʹ-CAG CAC GAG GCT TTT TTG TTG-3ʹ |
| **IL-6** | 5ʹ-ACA AGT CGG AGG CTT AAT TAC ACA T-3ʹ |  | 5ʹ-TTG CCA TTG CAC AAC TCT TTT C-3ʹ |
| **TGF-beta** | 5ʹ-GCA ACA TGT GGA ACT CTA CCA GAA-3ʹ |  | 5ʹ-GAC GTC AAA AGA CAG CCA CTC-3ʹ |
| **CCL2** | 5ʹ-GCT GAC CCC AAG AAG GAA TG-3ʹ |  | 5ʹ-GTG CTT GAG GTG GTT GTG GA-3ʹ |
| **CCL4** | 5ʹ-CCA GGG TTC TCA GCA CCA A-3ʹ |  | 5ʹ-GCT CAC TGG GGT TAG CAC AGA-3ʹ |
| **ALOX12** | 5ʹ-GAA GCC GGA CCC AGC TCA TC-3ʹ |  | 5ʹ-CTC AGC AGG CCT CGC TTA GC-3ʹ |
| **ALOX15** | 5ʹ-CGC CGA TTT TCA CGC CCT TG-3ʹ |  | 5ʹ-GGT GGC CAC AGC AAA GAC CT-3ʹ |
| **FPR2** | 5ʹ-TGG CTG GTT CCT GTG TAA AT-3ʹ |  | 5ʹ-CCA AGG CAA TGA GAG CAA TC-3ʹ |
| **MMP2** | 5ʹ-AAC TAC GAT GAT GAC CGG AAG TG-3ʹ |  | 5ʹ-TGG CAT GGC CGA ACT CA-3ʹ |
| **MMP3** | 5ʹ-GGA AAT CAG TTC TGG GCT ATA CG-3ʹ |  | 5ʹ-TAG AAA TGG CAG CAT CGA TCT TC-3ʹ |
| **MMP8** | 5ʹ-GAT TCA GAA GAA ACG TGG ACT CAA-3ʹ |  | 5ʹ-CAT CAA GGC ACC AGG ATC AGT-3ʹ |
