# Supplementary figures and images for: LXA4-FPR2 signaling regulates radiation-induced pulmonary fibrosis via crosstalk with TGF-β/Smad signaling
Source: Cell Death Dis. 2020 Aug 8;11(8):653. doi: 10.1038/s41419-020-02846-7 (PMC7434774; doi:10.1038/s41419-020-02846-7)

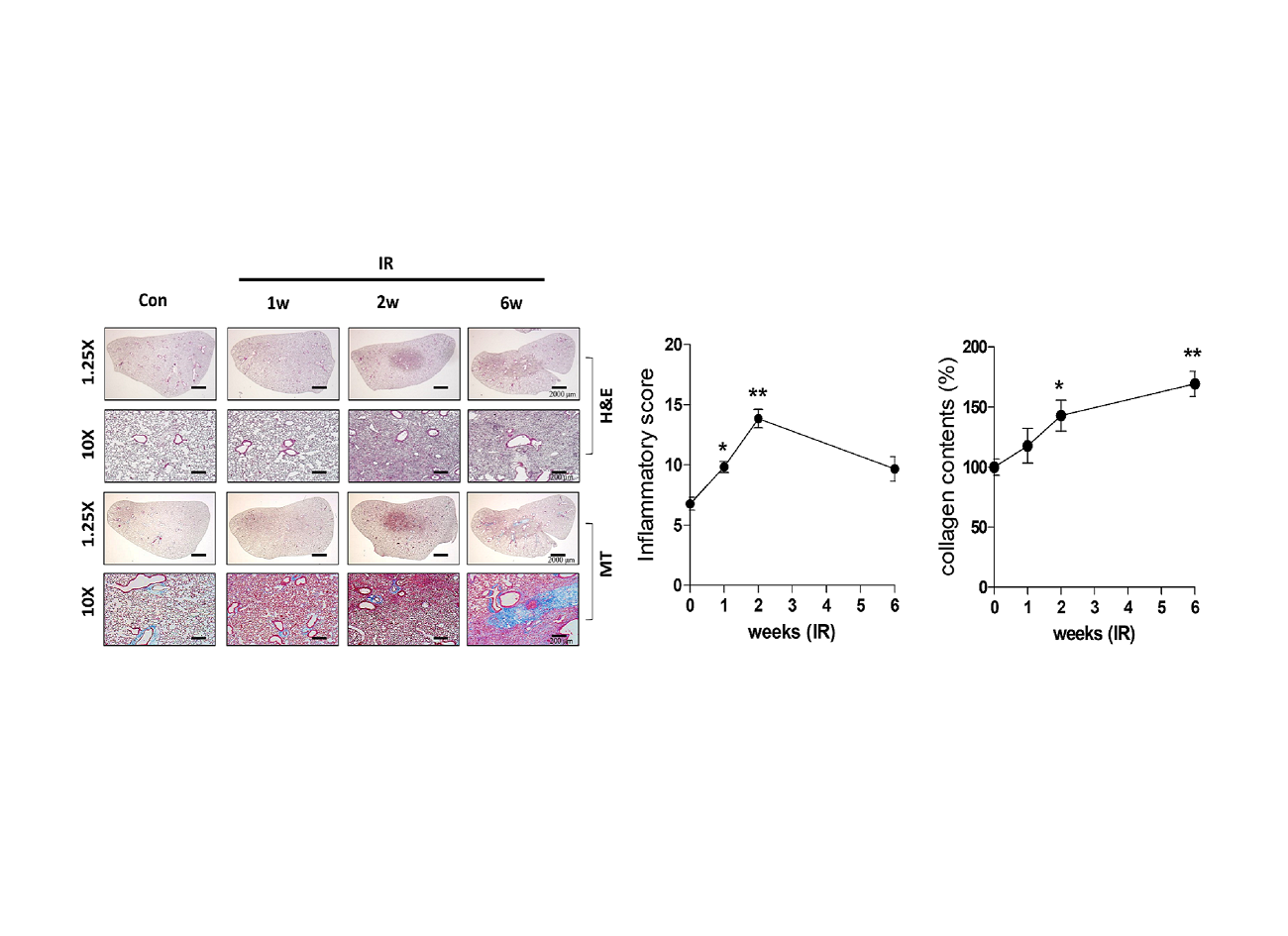

Supplement: Supplementary file 4 — Supplementary figure 1 [file 41419_2020_2846_MOESM4_ESM.tif]

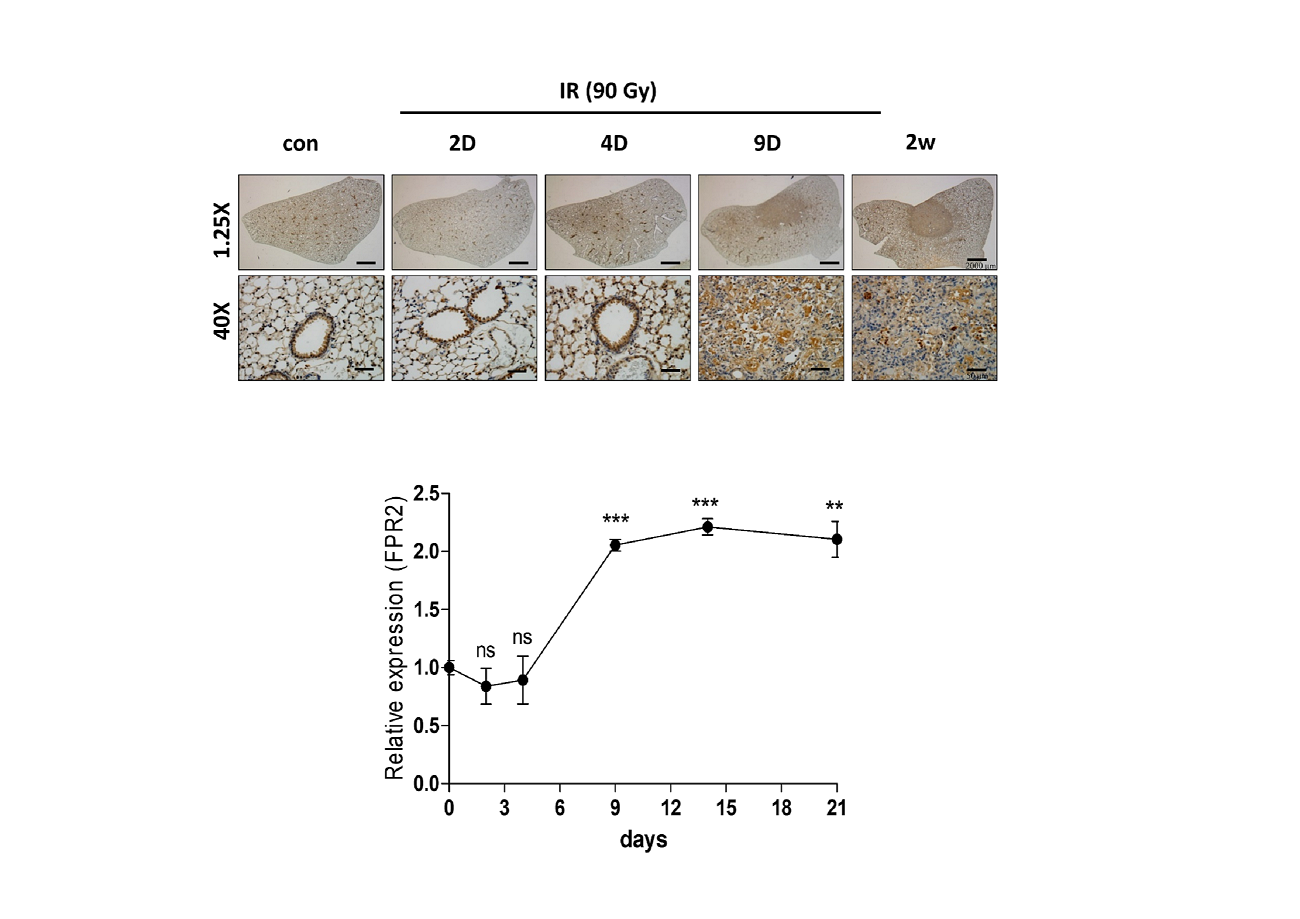

Supplement: Supplementary file 5 — Supplementary figure 2 [file 41419_2020_2846_MOESM5_ESM.tif]

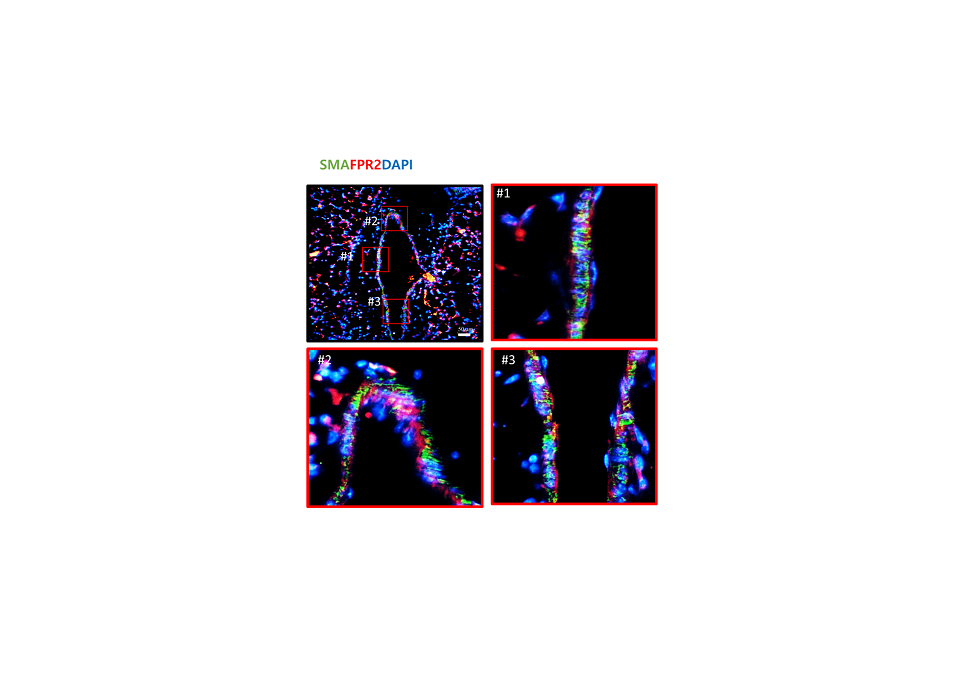

Supplement: Supplementary file 6 — Supplementary figure 3 [file 41419_2020_2846_MOESM6_ESM.tif]

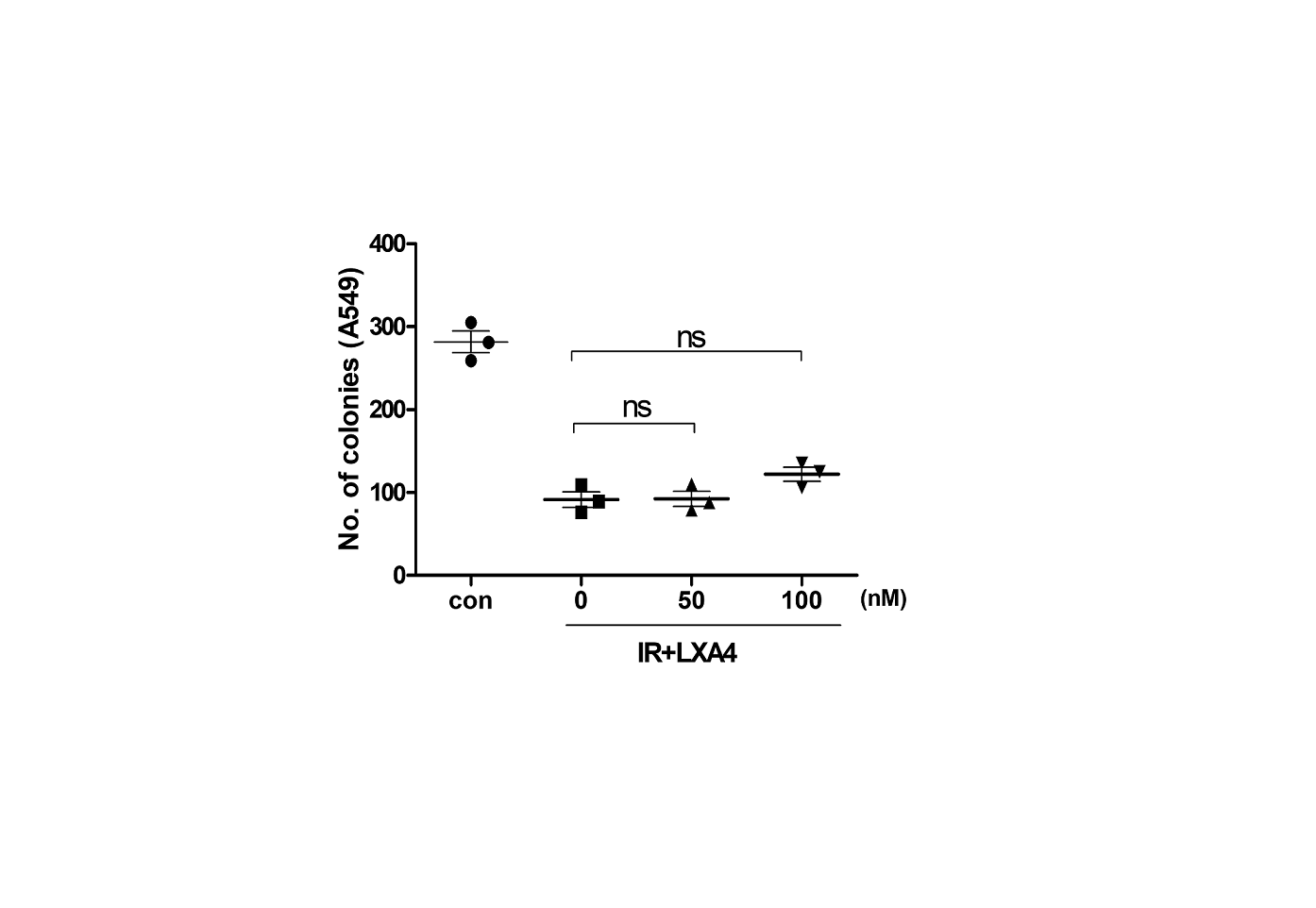

Supplement: Supplementary file 7 — Supplementary figure 4 [file 41419_2020_2846_MOESM7_ESM.tif]
